# Optimising and profiling pre-implementation contexts to create and implement a public health network intervention for tackling loneliness

**DOI:** 10.1186/s13012-020-00997-x

**Published:** 2020-05-19

**Authors:** J. Ellis, R. Band, K. Kinsella, T. Cheetham-Blake, E. James, S. Ewings, A. Rogers

**Affiliations:** 1 School of Health Sciences, Faculty of Environmental and Life Sciences, University of Southampton, Building 67, University Road, Southampton, SO17 1BJ UK; 2 Public Health Institute, Liverpool John Moores University, 3rd Floor Exchange Station, Tithebarn Street, Liverpool, L2 2QP UK; 3 School of Health Sciences, University of Southampton, Building 67, University Road, Southampton, SO17 1BJ UK

## Abstract

**Background:**

The implementation of complex interventions experiences challenges that affect the extent to which they become embedded and scaled-up. Implementation at scale in complex environments like community settings defies universal replication. Planning for implementation in such environments requires knowledge of organisational capacity and structure. Pre-implementation work is an important element of the early phase of preparing the setting for the introduction of an intervention, and the factors contributing towards the creation of an optimal pre-implementation community context are under-acknowledged.

**Methods:**

To explore the factors contributing towards the creation of an optimal pre-implementation context, a quasi-ethnographic approach was taken. The implementation of a social network intervention designed to tackle loneliness in a community setting acts as the case in example. Observations (of meetings), interviews (with community partners) and documentary analysis (national and local policy documents and intervention resources) were conducted. Layder’s adaptive theory approach was taken to data analysis, with the Consolidated Framework for Implementation Research (CFIR) and a typology of third-sector organisations used to interpret the findings.

**Results:**

Community settings were found to sit along a continuum with three broad categories defined as Fully Professionalised Organisations; Aspirational Community, Voluntary and Social Enterprises; and Non-Professionalised Community-Based Groups. The nature of an optimal pre-implementation context varied across these settings. Using the CFIR, the results illustrate that some settings were more influenced by political landscape (Fully professional and Aspirational setting) and others more influenced by their founding values and ethos (Non-Professionalised Community-Based settings). Readiness was achieved at different speeds across the categories with those settings with more resource availability more able to achieve readiness (Fully Professional settings), and others requiring flexibility in the intervention to help overcome limited resource availability (Aspirational and Non-Professionalised Community-Based settings).

**Conclusions:**

The CFIR is useful in highlighting the multiple facets at play in creating the optimal pre-implementation context, and where flex is required to achieve this. The CFIR illuminates the similarities and differences between and across settings, highlighting the complexity of open system settings and the important need for pre-implementation work.

**Trial registration:**

ISRCTN19193075

Contributions to the literature

- The importance of pre-implementation work to understand the context is well documented, but this paper goes further and illuminates the factors that are relevant to the implementation of a social network intervention designed to tackle loneliness in a community setting.
- Factors contributing towards the creation of an optimal pre-implementation community context are under-acknowledged in the literature, and this paper highlights the factors contributing towards the creation of an optimal pre-implementation community context ready for the implementation of a public health intervention.
- Intervention elasticity and context pliability are acknowledged to be important factors in the implementation process; this paper moves beyond elasticity and pliability in closed clinical settings by testing these in relation to a public health intervention being implemented in a community setting.

## Background

Key challenges are evident in the extent to which the implementation of complex interventions can become an embedded part of routine practice and are able to be scaled-up. There is increasing recognition that implementation at scale in complex environments is far from simple and defies a method for universal replication [1]. This is particularly the case for interventions destined for community settings. Interventions designed specifically to engage hard-to-reach groups are often reliant on organisations and informal settings which have the least capacity to deliver. Planning for implementation in Community, Voluntary and Social Enterprise (CVSE) organisations requires knowledge of organisational capacity and structure. These are frequently more hidden and less formulaic than those of the formal health care system, and therefore, this necessitates the need for pre-implementation work. The purpose of pre-implementation work is to identify the nature of the settings and to understand the fit between the intervention and the settings’ capacity to deliver in terms of reach, engagement and other markers of readiness. Readiness is a concept that refers to the extent to which those individuals who are likely to be involved in implementation can individually and collectively be primed and motivated. It refers to their capacity and capability of making change in a way which enhances or negates the implementation process [2–5].

Initial readiness becomes more important the more open the context. Open systems such as community settings are more entangled and messier than closed systems where entities and processes are independent from one another [6]. Interventions in the ‘real-world’ settings like community settings are characterised by ‘dynamic complexity’ of non-linear relationships between system elements [7]. This poses challenges for mapping the landscape and for the pre-implementation work that is required. In a review of the evidence-to-practice gap for complex interventions in primary care, the degree of preparation prior to implementation was identified as a key element of successful implementation [8]. Similarly, the early phase of preparing the ground for the introduction and testing of public health interventions has been demonstrated as a key facet of large-scale improvement [9].

There is an increasing focus on examining how interventions interact with the flux of changing contexts [1, 3, 10]. Pfadenhauer et al. [11] view context as the set of active unique characteristics and circumstances surrounding implementation that hold the capacity to modify, facilitate or inhibit the implementation of an intervention*.* Complex systems as in the case of open systems are characteristically unpredictable, unstable and constantly unfolding [12], and they adapt, interact and co-evolve with other local systems [1]. Viewing context and implementation in an integrative way coalesces with the notion that flexibility is required to support implementation and that alignment between context and intervention is achieved with flexibility within each component [13].

Engaging in pre-implementation work offers opportunities for tracking this evolution, the results of which can be an aid to encourage successful implementation and sustainability [13]. Our focus here is on better understanding the organisational arrangements and work required prior to the implementation of an intervention. This is taking place in a landscape that is moving towards the CVSE sector playing a greater role in the health and social care strategy [14], which interface with yet another layer of complexity, that is, the complexity arising from the context needs at the intersection with the resources and networks operating in peoples’ everyday lives [15].

The specific example we are concerned with here is the implementation of a social network intervention designed to tackle social isolation and loneliness by connecting people to the resources in their community allowing them to take part in activities or utilise support. In the UK, 30% of the adult population experience social isolation or loneliness [16], with studies reporting a negative impact on physical and mental health outcomes [17]. Studies have highlighted how connecting to community resources can help protect against loneliness [18] and that improving the quality of existing relationships as well as increasing participation in activities may help tackle the impact of loneliness and social isolation [19]. Thus, it follows that implementing a social network intervention in the CVSE sector to connect people who are at risk of social isolation or loneliness could help address this public health concern.

The Project About Loneliness and Social networks (PALS) is a hybrid designed [20] pragmatic randomised controlled trial (RCT) (the study protocol is available [21]). The hybrid design simultaneously assesses the effectiveness and cost-effectiveness of a facilitated social network intervention (Genie intervention), as well as the implementation of this in a community setting through an embedded process evaluation. The intervention has been developed and tested in the context of managing long-term health conditions [22–24]. However, the intervention has yet to be tested in relation to addressing loneliness, and it has yet to be implemented in CVSE environments. Thus, in order to maximise successful implementation, and ultimately the sustainability of the intervention in an open system, the logic and complexity of the CVSE environments within which the implementation team is working needs to be understood. Through the above acting as a case in example, this paper will explore the nature of community settings.

### Aim

This study examines the factors contributing towards the creation of the optimal pre-implementation context. The study also examines the extent to which CVSE environments and people within it need to adapt to allow space for introducing the intervention (elasticity), and the extent to which the intervention elements be moulded to accommodate the context (pliability).

## Design

A quasi-ethnographic approach was used because it affords opportunities for different and hidden aspects of the settings to be revealed [25]. Data collection occurred from May 2018 and continues throughout the duration of PALS (end March 2021). Ethical approval was granted by the South Central Berkshire Health Research Authority and the University of Southampton Research and Governance Ethics Committee prior to data collection.

### Study setting

PALS is being delivered in collaboration with community partners in Southampton and Liverpool, UK. Partnerships were sought with any organisation or group who works in the community with the potential to identify or access individuals at risk of social isolation or loneliness [21]. Potential partners were either purposively contacted by the implementation team or referred in by a process of snowballing. A total of 22 partners were recruited (Table 2). The partners fall on a continuum from statutory service providers (services required by law, i.e. police, fire brigade, ambulance) to places of religious worship, health centres, branches of national charities, local social enterprise start-ups and community associations that fall in between.

**Fig. 1:**
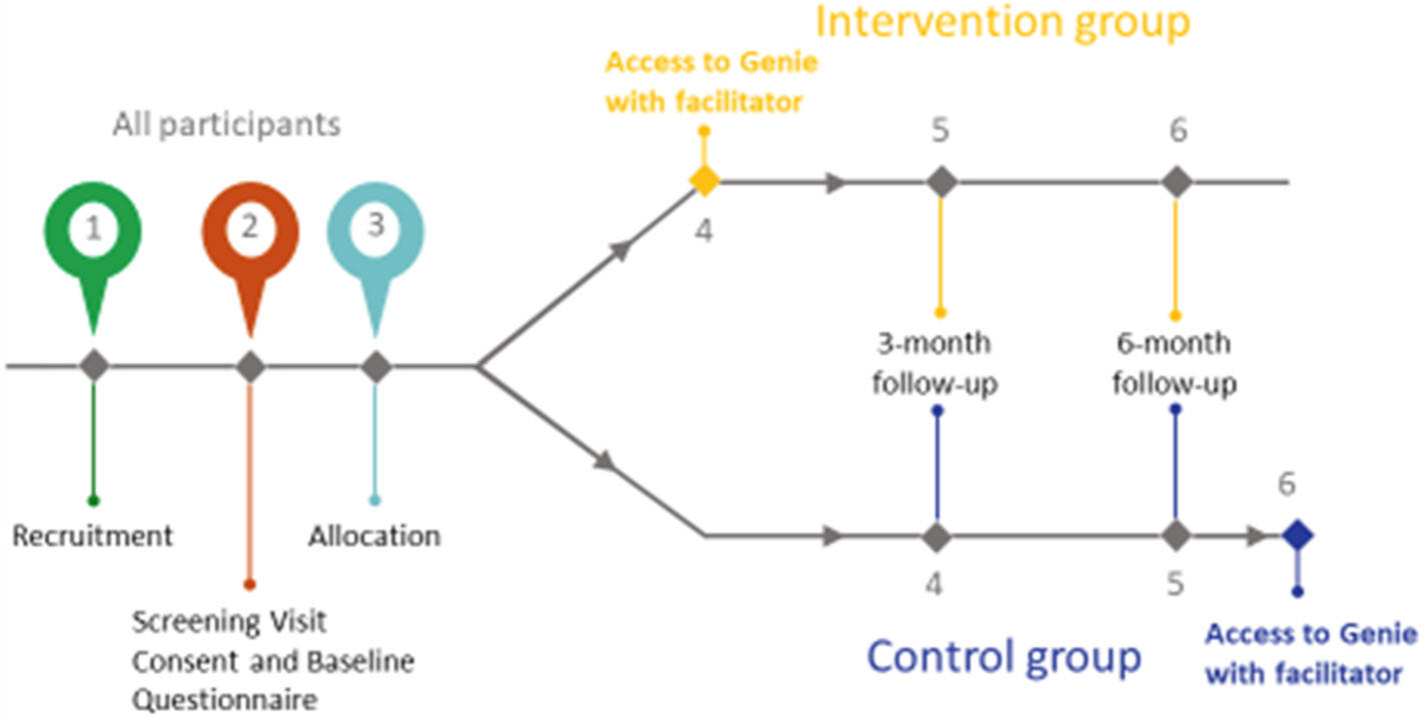
Workflow

Each partner represents an implementation site, a study setting. The implementation team and the partners worked to deliver the RCT, and the division of labour is illustrated in Fig. 1.

The partners were responsible for the identification of intervention facilitators to receive training in delivering Genie, the identification and recruitment of participants, the delivery of Genie (to the intervention group, within 2 weeks following consent; to the control group, following a 6-month questionnaire) and a follow-up 3-month social network mapping exercise (intervention group only). The ‘work done’ by the implementation team included facilitator training, taking participant consent, baseline data collection and 3- and 6-month follow-up data collection.

### Methods

The quasi-ethnographic approach allowed for immersion and for an iterative approach to data collection. Firstly, documents were gathered from the outset of the study. This included the study protocol and training material to better understand the implementation team’s desires. To help understand the broad political landscape, local authority health and wellbeing reports and strategies were collected. These were publicly available from the local authorities’ websites, as well as leaflets and materials produced by partners to help understand their context.

Secondly, occurring continually throughout and contributing towards the bulk of the data, observations were made by JE. Assuming the dual position of a team member and a researcher, JE was able to observe the implementation team in a way not afforded to an outsider. Observations were made on the implementation team’s internal meetings and email communication to understand the team’s position and reaction to the implementation endeavours. To understand partners’ contexts and the potential impact of the study on the contexts, the meetings between the implementation team and partners were observed. All notes were made in situ on matters around organisational features, readiness and intervention fit, as well as the pliability of the intervention.

Thirdly, occurring simultaneously, interviews were carried out with representatives from the partners and were between 30 and 60 min in length. All organisations were approached and invited to interview via email/call, and in total, 14 interviews were conducted across 11 partners and included managers and employees (potential Genie facilitators). An interview guide was developed in conjunction with the framework (described below) and discussions with the implementation team (additional material). In keeping with the ethnographic approach, this was used flexibly, and topics varied as points of interest arose. Interviews were carried out in person or by phone by JE, KK or TCB and were audio-recorded and transcribed verbatim. Reflective notes were made after each interview to capture non-verbal elements.

### Data analysis

Data analysis was informed by Ladyer’s [26] adaptive theory; this allowed for an iterative approach to data analysis. The process of familiarisation, coding (inductively and deductively) and interpretation continued throughout to help ground onward data collection. JE performed these tasks, reporting back to the team regularly to sense check, before working collaboratively with AR to interpret the findings. Coding deductively drew upon the Consolidated Framework for Implementation Research (CFIR) [3] and through a process of inductive coding, Buckingham’s typology of third-sector organisations was used to interpret the findings.

The CFIR outlines five key domains that interact to influence implementation. The five domains have 37 sub-domains combined and are listed in Table 1. The CFIR was a suitable framework as it was useful in helping to understand the factors important to pre-implementation work.

**Table 1:** CFIR

CFIR domain	Sub-concept
Intervention characteristics	Intervention source Evidence strength and quality Relative advantage Adaptability Trialability Complexity Design quality and packaging Cost
Outer setting	Patient needs and resources Cosmopolitanism Peer pressure External policies and incentives
Inner setting	Structural characteristics Networks and communications Culture Implementation climate (relative priority, organisational incentives and rewards, goals and feedback, learning climate) Readiness for implementation (leadership engagement, available resources, accessible information and knowledge)
Individual characteristics	Knowledge and beliefs about the intervention Self-efficacy Individual stage of change Individual identification with organisation Other personal attributes
Process of implementation	Planning Engaging (opinion leaders, formally appointed internal implementation leaders, champions, external change agents) Executing Reflecting and evaluating

Buckingham’s [27] typology of third-sector organisations was used to inform the interpretive section and to guide understanding of the nature of the partner’s settings. The four typologies included the following: comfortable contractors who operate at the national and regional scales are involved in government contracts and have no volunteers or voluntary income; compliant contractors are regional-level providers who are heavily dependent on government contracts and have little volunteer involvement and income, and they are described as charities that have become professionalised; cautious contractors have some involvement in government contracts but for whom the voluntary income is significant and have a workforce of paid staff and volunteers; and community-based non-contractors are not involved in government contracts and are dependent on voluntary income, are embedded in the local community and reliant on volunteers.

## Results

The nature of the community partner’s context was found to influence the pre-implementation work required. Buckingham’s [27] typology was a useful starting point for better understanding the range of contexts of operation. The partners were categorised from the information they had provided in the interviews and from documents gathered. What was found is that the community partners did not fall discreetly into a typology. Rather, what was found to exist was a continuum of contexts blending more smoothly from being more comfortable into more compliant, into more cautious contractors and into more community-based non-contractors. Few partners fell wholly into a typology, and where this did occur, it was often at the extreme ends: comfortable contractors (i.e. statutory service providers) and community-based non-contractors (i.e. places of worship). Many partner contexts displayed elements of two typologies, and so to reflect this continuum, three categories have been defined to reflect the clusters of partners falling on the continuum of typologies, as well as being useful to account for the complexities in settings. Table 2 describes the partners, places them on the continuum and illustrates where the three new categories overlap with Buckingham’s typology.

**Table 2:** Community partner continuum

Typology	Number	Community partner description	Category
Comfortable contractors	1 2 3 4 5 6 7 8	Statutory service provider Statutory service provider Collection of GP practices Statutory service provider Health centre Community interest company Research centre Housing association	Fully Professionalised Organisations
Compliant contractors	9 10 11 12 13 14 15	International charity—local branch Group of charitable social enterprises Group of charitable projects Charity—city-based National charity—local branch National charity—local branch Charity—city-based	Aspirational Community, Voluntary Social Enterprise
Cautious contractors	16 17 18 19 20	Charity—city-based Charity (formed from merger) Charity (formally faith-based organisation) Community association Community centre	
Community-based non-contractors	21 22	Place of worship—church Place of worship—mosque	Non-professionalised Community-Based Groups

To unpick what contributes towards the optimal pre-implementation context, and the nature of pre-implementation work, the findings take the newly formed categories as the basis of structuring the discussion: Fully Professionalised Organisations, Aspirational CVSEs and Non-Professionalised Community-Based Groups. The letters I, O and D are used to label the data excerpts from interviews, observations and documents, respectively, and the partner (P) numbered in correspondence to Table 2.

### Fully Professionalised Organisations

Fully Professionalised Organisations (FPOs) are very business-minded; these highly professionalised settings often deliver services over a large geographical area and may even operate nationally with localised hubs. Importantly, in FPOs, there is a formal hierarchical structure and bureaucracy. Characteristically, they have secure contracts with local authorities and are competitive and entrepreneurial in nature.

These partners were heavily influenced by the political landscape because they were entangled with, embedded within and reliant upon government contracts. They themselves acknowledge they are ‘very politically swayed’ in the design of their ‘corporate strategy’ (IP8). As such, where there was close alignment between the intervention and the political agenda, captured by the CFIR sub-concept ‘external policies’, this was influential in securing buy-in from the leadership of these partners. In this example, loneliness had recently been declared a public health risk [28]; nationally, the NHS Long-Term Plan [14] outlined the importance of social prescribing and community assets in delivering universal personal care. Thus, for managers of FPOs, the political climate was heavily influential in securing buy-in, and therefore, the sub-concept ‘external policies’ becomes important in creating the optimal pre-implementation context. This was seen in the example of an early engagement meeting with representatives from several GP practices.

We were introduced to the room with some level of enthusiasm saying we were there to talk about linked to the new commissioning brief and the push for increased social prescribing that the navigators were to do (OP3)

In instances where the policy context and intervention aligned, managers saw the ‘relative advantage’ of the opportunity to trial the intervention that could help fulfil a strategic objective. The sub-concept ‘leadership engagement’ was also relevant in this context across all partner contexts. By securing ‘leadership engagement’ and with the intervention offering ‘relative advantage’ (i.e. fulfilling a strategic objective), these partners were more willing and able to allocate resources to aid implementation. This was particularly so in terms of human resources; in instances, this was clear ‘PALS was written into our job specifications, it is an objective for us’ (IP8), and in other instances, there was a clear commitment ‘we’ll find ways for this to work because it does overlap with what we do normally’ (IP3). This commitment helped to create a climate encouraging of implementation and one with sustainability potential due to the ‘resources available’. The ability to replace intervention facilitators easily to ensure implementation continued is an example.

[Female] going on MAT [maternity] leave in June, but as we were leaving the office we were introduced to [Male] who will replace [Female] as the facilitator. PALS had already been written into his objectives for the next quarter. (OP8)

With the potential to create a climate encouraging of implementation, the pre-implementation context was also contingent on factors beyond ‘political landscape’ and ‘leadership engagement’.

One factor relates to the sub-concept ‘patient needs’. In many instances, FPOs deliver services to individuals who are ‘in crisis’ (OP3) and who are ‘dealing with the immediate’ and often very complex issues (OP8). Where individuals are dealing with profound problems, partners also reported the individual can become ‘easily overwhelmed’ (IP4). This speaks to the suitability and timing of the intervention being implemented. In this example, some of the ‘patient needs’ were perceived to be too severe and complex, which led some partners to view Genie as being unsuitable for participants.

Although ‘leadership engagement’ was influential in the creation of the optimal pre-implementation climate and also ‘implementation readiness’, those responsible for delivering the intervention were in the beginning often excluded from engagement conversations, and yet were integral to implementation. In instances where there was ‘leadership engagement’, the pre-implementation context was still negatively affected where buy-in failed to be secured at all levels. As illustrated in this example, buy-in had been secured at the senior management level, but middle management and those identified as potential facilitators had not been consulted about the partnership.

This is benefiting the service, but they [the potential facilitators] see people with real difficult psychological problems who are in immediate threat, there’s safeguarding and real difficult stuff. Loneliness is important, and I’m not saying it isn’t, but is it of the same standard. Can I justify their time? (OP4)

Relating to the sub-concept ‘knowledge and belief about intervention’, failure to acquire the insights across the workforce hierarchy led to the need to troubleshoot further down the line, which also illustrates the need for pliability (as the intervention team worked flexibly within the protocol to tailor the division of labour to each partner). Through a process of monitoring and reflection, the need to secure multi-level buy-in (rather than assume organisations would internally secure this) became a priority and was solved by the introduction of two meetings. One meeting took place ahead of facilitator training, and one that took place after the facilitators had been trained. These meetings took place with everyone directly involved in the implementation process and often included a member of leadership, middle management (who tended to have direct oversight of the project) and individuals becoming intervention facilitators. These meetings were important for all contexts but especially so for the FPOs where the workforce tended to be larger, and there was more risk of a disconnect between top-level managers and those responsible for delivery. These meetings enabled troubleshooting to take place earlier and ensure that buy-in was secured from all involved. The meetings were a real contributor to the creation of coherence [29] and thus the optimal pre-implementation context.

Where the necessary elements were in place to create the optimal pre-implementation climate, readiness for implementation was time-sensitive. Being business-like in operation, where there are objectives to deliver on within a time frame, often meant the organisations’ pace of work was the fastest of all categories. Thus, these partners reported that in their context, ‘it is a changing picture, when we say go you go or you’ve missed it’ (IP8), and any delay from the implementation team may mean the window of readiness closes. Whilst the experience here was that the window was never closed completely, some delays on the implementation team due to the research side of the project regarding ethical administration (vs intervention readiness) did mean moments of readiness for some partners were missed. As in the example where changes to the ethical approval process stalled implementation, and where facilitators had been trained, the delay meant that the facilitators required refresher training.

To be honest things are really busy leading up to Christmas so a refresher training for the team would have to happen in the new year (OP9)

This illuminates the different challenges posed by the intervention and by the research trial; in this example case, it was often the latter posing the challenges. However, it serves as a caution to readers that when working in open systems, but especially in FPOs, readiness is paramount on both the intervention and the research side. The optimal pre-implementation context has several contributing factors, and the window of implementation readiness may be fleeting.

### Aspirational CVSE

Aspirational CVSEs are an amalgamation of the compliant and cautious contractors [27]. It refers to those CVSEs who mainly operate at a regional to a local level and for whom voluntary income and volunteer involvement are important contributors. Aspirational CVSEs tend to try to stay as close to their values and ethos and have a small structured workforce.

Of all partners, these partners balance being influenced by ‘external policies’, particularly the local political agenda, and being authentic to the values on which they were founded. Aspirational CVSEs further along the professionalisation journey were more influenced by ‘external policies’, as ‘the local policy arena dictates some procedures, but it is not the only driver’ (IP10). Whilst for the less professionalised partners, staying close to founding values and ethos was more important.

There are some reservations I have about whether it [Genie]…we don’t want to become a clipboard organisation (IP18)

For Aspirational CVSEs, if the intervention aligned with their main influence, either ‘external policies’ or founding values, or the tension between the two had been reconciled, ‘leadership engagement’ was secured. Like FPOs, this was an important contributor towards creating the optimal pre-implementation context. Furthermore, ‘leadership engagement’ was also influenced by the ‘relative advantage’ of the intervention and the benefit to be gained. Here, it was the potential of receiving an evaluation of their services, especially so where alignment exists, and the intervention was considered to be ‘a formalised version of what we do with people who use our service’ (OP16).

I want to be able to measure the impact of the work, the service, I want evidence about what we do. (OP16)

The second ‘relative advantage’ was the potential participation had in ‘upskilling the team’ (IP10). This was of particular interest to the more professionalised partners, whereas the potential to offer evidence was more of interest to the less professionalised Aspirational CVSE partners. These ‘relative advantages’ were significant pulls because of the precarious tenure of partners. Thus, ‘leadership engagement’ was influenced by the potential evidence the research component could provide, enabling Aspirational CVSE partners to utilise this to support future funding applications.

The more precarious a partner’s tenure, the more this influenced ‘leadership engagement’. This was because leaders considered the scarcity of ‘resource availability’. Perceived ‘resource availability’ was in some ways the most significant contributor of the pre-implementation context. Part of the pre-implementation work when seeking to implement an intervention in Aspirational CVSEs concerned troubleshooting human, financial and physical resource availability, especially for the least professionalised partners, as in the example of partner 18 who outlined ‘at the moment we’re very short-staffed, I don’t think we have the capacity in our paid staff to do it [Genie] through them’ (IP18). This limited human resource availability meant there was less flexibility, specifically because the same workforce was required to do several jobs. This posed challenges for the research element of the partnership and required pliability. In response, the implementation team worked flexibly within the stated protocol to maintain methodological rigour but also accommodate the human resource limitation. This pliability was required in order to accommodate the context and promote alignment but ensure methodological rigour.

Aspirational CVSEs were more insecure than the FPOs. These partners also experienced high demand for their services with shrinking budgets; thus, capacity was stretched and the flexibility within these settings was limited compared to the FPOs settings. Take for example the pivotal role of volunteers. Where the intervention is an additional task for a setting already at capacity, the role of volunteers may be essential to the implementation, as in the case of partners 14 and 18. The additional difficulty of implementing an intervention that requires the use of technology placed further strain on resources and on budgets where there was no flexibility to accommodate this. Pliability was required to support the maximising of available resources, for example, finding volunteers as in the case of partner 18 or supplying physical resources to support intervention (i.e. laptops). Where the necessary resources can be mobilised, this makes for an encouraging pre-implementation context.

Overcoming the challenge of capacity that troubles many Aspirational CVSEs is important for implementation. It is also important for similar public health interventions that require self-directed change especially because there appeared to be close alignment between the ‘patient needs’ and the intervention.

We’ll use the information and support group, because the clients would be more able to go to the group (OP14)

Where FPOs provided support to people referred into the service due to being in crisis, Aspirational CVSE partners served a wider breadth of people often ‘picking up the people who fall through the gaps’ (IP18). These settings provide needed support to people in the community, and often, the people accessing the services were perceived as being suitable for the intervention. Individuals were not necessarily in crisis, and their needs tended to be less complex and multifaceted than those individuals accessing FPOs. This contributed towards an optimal pre-implementation context for there was a potential pool of participants for the research trial, but importantly, the Aspirational CVSE partners had access to people who were perceived to have potential to benefit from the intervention.

With the ability to access people with optimal needs (to the intervention), with ‘leadership engagement’ and with the challenge of ‘resource availability’ overcome, implementation readiness was achieved. For Aspirational CVSEs, the pace of achieving this readiness was slower than that of the FPOs; however, the potential for readiness stayed open for longer.

### Non-Professionalised Community-Based Groups

There were two Non-Professionalised Community-Based (NPCB) groups, which are those Buckingham [27] describes as community-based non-contractors, usually faith-based groups as these case studies are. These informal groups are embedded within the local community in which they are based. They offer activities which are not eligible for funding and rely heavily on voluntary donations and volunteers. These groups emphasise acceptance, promote community cohesion and are a site of sociability and often solidarity [27].

In both cases, the engagement with the project and the pre-implementation work was more relational than any of the other partners. In the first instance, access to the groups was negotiated by a network referral. Where in the more structured setting of Fully Professionalised and Aspirational partners initial contact was made with the managerial team, NPCB groups operate more informally and do not have such a formalised structure. Thus, the ‘way in’ to these settings may appear a little trickier, or even fortuitous, but it arose with a significant effort in relational work. That is, in-roads were made indirectly through a process of engagement with members of structured organisations such as local authorities, county councils and to a degree also with FPOs who often also have a high degree of cosmopolitanism. Through engaging with these highly networked members, access to NPCB groups was obtained via referral.

Hi [Name], I would like to introduce you to Becky who is leading on the PALS study. I will leave Becky to link with you to look at the opportunities… (OP21)

In these case studies of NPCB groups, this personal referral was to an individual who themselves were highly networked and embedded within the community. The role and importance of this individual in the pre-implementation work in championing the partnership were more integral than in any other setting. The champion negotiated between the implementation team and the group to achieve ‘leadership engagement’ (i.e. senior religious leaders).

I’ve now had the chance to talk with my colleagues at the church and they’re all very interested (OP21)

Unlike the other partners, the ‘outer setting’ of the CFIR was of less influence in securing buy-in. Of most importance was the sub-concept ‘culture’ of the ‘inner setting’, specifically of most influence was the values of the groups, that is, the values of promoting community cohesion and being a place of inclusion for all, including the congregation and members of the geographical community. Where the intervention was perceived to align with these values, i.e. was deemed to be beneficial to the community, this led to a commitment from the leadership and strengthened the champion’s resolve to implement Genie, as in partner 22 who considered PALS ‘an excellent project [that] could be very beneficial to our community therefore they were ‘at your disposal’ (IP22). The integral role of the champion in these settings cannot be overstated. However, the reliance on one individual, as in both cases here, due to limited ‘resources availability’ did contribute towards achieving readiness extremely slowly. Despite low resource availability and thus low absorptive capacity within groups, the commitment to support the community was the primary motivator, and even though the readiness for implementation was slower to achieve, the motivation remained, with the metaphor ‘I am like a tortoise…but hopefully we’ll get there in the end’ (IP21) being a fine example. NPCB groups were influenced and negatively impacted by limited resources. They were most influenced by the commitment to serve and support their local community, both the immediate congregation and the geographical community. Without such devotion of a champion, however, it is reasonable to question whether readiness for implementation would have ever be achieved.

## Discussion

The need to understand how public health interventions, (such as Genie), can successfully be implemented, embedded and sustained in community settings is relevant to executing contemporary health and social care initiatives (e.g. social prescribing in the UK) where these settings are looked at to provide psychosocial support to people. This paper has examined the nature of the optimal pre-implementation context and the arrangements that contribute towards this (Table 3 provides a summary), and in doing so illustrates how the complex and differing settings found in open systems demand different levels of pliability of the intervention.

**Table 3:** Key factors affecting the optimal pre-implementation context

CFIR concept	FPO	ACVSE	NPCB
Relative advantage	+	+	
Patient needs and resources	−	+/−	+
External policies and incentives	+	+/−	
Readiness for implementation (leadership engagement)	+	+	+
Readiness for implementation (available resources)	+	−	−
Knowledge and beliefs about the intervention	−		
Culture		+/−	+

Plus sign (+), positive impact/minus sign (−), negative impact

We found that that the CFIR is a useful framework for understanding the work required to achieve the optimal pre-implementation climate in open system settings. The CFIR illuminates the multiple facets at play and where flexibility is required and can be achieved in the most structured of organisations to the most informally constituted groups, in terms of influencing readiness for implementation and can be used to ascertain estimates regarding which setting has the maximum chances of successful implementation. Of relevance for the more formally structured organisations were the sub-concepts ‘leadership engagement’, ‘resource availability’ and ‘patient needs’. Across all setting types, leadership engagement was integral to being able to begin the pre-implementation work. This finding is consistent with others [30, 31] and adds to the literature by demonstrating the importance of leadership engagement for successful implementation across the different settings found in open systems, with leadership engagement flexibility within settings was more easily found. This is important as lack of elasticity and available resources in a setting can impact negatively on implementation and ultimately sustainability [13].

The CFIR was useful at illuminating the similarities and differences between and across settings, and in doing so, highlighting the complexity of open system settings. Specifically, although presented as three categories, settings actually fall along a continuum and have ‘more or less’ elements of the three presented categories. Settings displaying more Fully Professionalised Organisations and Aspirational elements were the most affected by the political landscape, which would impact upon resource availability and leadership engagement. Yet, the political landscape impacted upon all settings found within the open system in one way, as the impact of the UK’s austerity agenda of the last 10 years was seen to affect capacity. The more Fully Professionalised leaning settings appeared most resilient to the impact of austerity (although they were not immune), whilst NPCB leaning settings appeared most resolute to achieving implementation to combat the effects of austerity and ‘make a difference’. Yet, it was the impact on the capacity of more Fully Professionalised and Aspirational CVSE settings that demanded pliability (seen in the need for the intervention team to work flexibly within the stated protocol and assist in the recruitment of volunteers). MacMilan and Ellis-Pain’s [32] observed that budgetary constraining on front line work of community organisations is highly pressurised, positing questions regarding future implementation and sustainability. Within this political landscape, all settings are having to be more creative and pioneering [27] in order to be more competitive to ensure their own survival [32], which perhaps contributed towards the fuzzy boundaries between settings, as settings try to adapt to survive.

The findings illustrate the contextual demands when implementing in open systems, highlighting the elasticity achievable in each context and the pliability required of the intervention. Furthermore, they support the literature regarding how failure to engage with complexity can lead to non-adaptation or abandonment [33], as well as illustrating further support for the need to see an intervention as a negotiated entity that requires ongoing work to fit the context to help implementation and sustainability [13].

### Implications and study limitations

The implications drawn from this study relate to Greenhalgh et al.’s [34] call for studies to be more locally situated. Specifically, here, through the use of the CFIR, the need to be contextually sensitive and locally situated in setting up an RCT in the community can be seen and better understood. Implications also extend to highlight the importance of time and relational work in implementation. That is, when implementing in Fully Professionalised Organisations, the relationship is more transactional, and when readiness is achieved, it may be fleeting. Thus, the implementation team should have all aspects ready to move on demand. For settings displaying more Aspirational and NPCB elements, more time is required to build relationships and determine elasticity and pliability. Thus, the pre-implementation arrangements take longer to organise, and this process should start sooner and not necessarily wait for the implementation team to be ‘ready’. We acknowledge that the current findings are limited to the implementation of a public health intervention within a UK community context and therefore should be interpreted accordingly.

## Conclusions

The concluding thoughts turn to issues of sustainability. Where Fully Professionalised Organisations are more secure in tenure and have resources, they are heavily reliant upon the political landscape staying aligned to that of the intervention focus. This raises questions around the sustainability potential of an intervention that is not in political favour. The more Aspirational CVSEs place greater emphasis on remaining true to their values rather than political landscape, but have less resource capacity, which poses the question, Are they more likely to continue with an intervention if its focus is less of a political ‘hot topic’, and importantly, do they have the capacity to? Future studies will go on to explore how sustainability is affected when greater understanding of how settings affect pre-implementation arrangements is achieved.

## Data Availability

The datasets during and/or analysed during the current study are available from the corresponding author on reasonable request.

## Abbreviations

CVSE: Community, Voluntary and Social Enterprise
PALS: Project About Loneliness and Social networks
RCT: Randomised controlled trial
Genie: Generating Engagement in Network Involvement
CFIR: Consolidated Framework for Implementation Research
FPO: Fully Professionalised Organisations
NHS: National Health Service
